# HIF‐1α promotes astrocytic production of macrophage migration inhibitory factor following spinal cord injury

**DOI:** 10.1111/cns.14300

**Published:** 2023-06-19

**Authors:** Huifei Hao, Yuxuan Hou, Aicheng Li, Li Niu, Shaolan Li, Bingqiang He, Xingyuan Zhang, Honghua Song, Rixin Cai, Yue Zhou, Chun Yao, Yongjun Wang, Yingjie Wang

**Affiliations:** ^1^ Key Laboratory of Neuroregeneration of Jiangsu and Ministry of Education, Co‐Innovation Center of Neuroregeneration Nantong University Nantong China

**Keywords:** astrocyte, HIF‐1α, hypoxia, inflammation, MIF, spinal cord

## Abstract

**Background:**

Macrophage migration inhibitory factor (MIF) is an important mediator of neuropathology in various central nervous system (CNS) diseases. However, little is known about its inducers for production from the nerve cells, as well as the underlying regulatory mechanism. Injury‐induced HIF‐1α has been shown to exacerbate neuroinflammation by activating multiple downstream target molecules. It is postulated that HIF‐1α is involved in the regulation of MIF following spinal cord injury (SCI).

**Methods:**

SCI model of Sprague–Dawley rats was established by cord contusion at T8–T10. The dynamic changes of HIF‐1α and MIF protein levels at lesion site of rat spinal cord were determined by Western blot. The specific cell types of HIF‐1α and MIF expression were examined by immunostaining. Primary astrocytes were isolated from the spinal cord, cultured and stimulated with various agonist or inhibitor of HIF‐1α for analysis of HIF‐1α‐mediated expression of MIF. Luciferase report assay was used to determine the relationship between HIF‐1α and MIF. The Basso, Beattie, and Bresnahan (BBB) locomotor scale was used to assess the locomotor function following SCI.

**Results:**

The protein levels of HIF‐1α and MIF at lesion site were significantly elevated by SCI. Immunofluorescence demonstrated that both HIF‐1α and MIF were abundantly expressed in the astrocytes of the spinal cord. By using various agonists or inhibitors of HIF‐1α, it was shown that HIF‐1α sufficiently induced astrocytic production of MIF. Mechanistically, HIF‐1α promoted MIF expression through interaction with MIF promoter. Inhibition of HIF‐1α activity using specific inhibitor markedly reduced the protein levels of MIF at lesion site following SCI, which in turn favored for the functional recovery.

**Conclusion:**

SCI‐induced activation of HIF‐1α is able to promote MIF production from astrocytes. Our results have provided new clues for SCI‐induced production of DAMPs, which may be helpful for clinical treatment of neuroinflammation.

## Graphical abstract

HIF‐1α promotes astrocytic production of MIF in astrocytes following SCI.

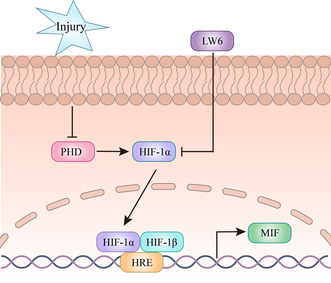

## 1 BACKGROUND

Traumatic injury to the spinal cord always results in the loss of locomotor function, which is worsened by, to some extent, a complex cascade of secondary injury events.^1^ At the early acute injury phase, the neurons, astrocytes, and oligodendrocytes at lesion site suffer to damage, in conjunction with the activated immune cells and the disrupted vasculature and the blood–spinal cord barrier (BSCB).^2^ Immediate after that, a more insidious and deleterious secondary tissue damage is programmed to arrive and persists for several days or longer, characterized by ischemia, cellular necrosis, formation of glial scar, and cystic cavities.^3^ The overwhelming inflammatory response evoked by damage‐associated molecular patterns (DAMPs) is recognized as a culprit in initiation of the pathogenesis, as genetic deletion or pharmacological inhibition of endogenous DAMPs or the according receptors has been shown to improve the locomotor function.^4^ Injury‐induced DAMPs are derived from active secretion by immune‐related cells or passive release from necrotic cells.^5^, ^6^ However, the specific factor(s) that drives the production of DAMPs from resident nerve or immune cells, as well as the underlying mechanism following SCI remains unclear.

Astrocytes are the abundant glial cells that widely distribute in the central nervous system (CNS), associating with health and disease.^7^, ^8^ They have a myriad of well‐documented roles in support of neuronal homeostasis including formation of the blood–brain barrier, secretion of the neurotrophic factors and control of the microenvironment.^9^, ^10^ In response to stimuli, astrocytes can undergo morphological, molecular and functional remodeling termed reactivity, which can exert a protective or detrimental influence on surrounding neurons.^11^, ^12^ The reactive astrocytes are also considered as active player in the CNS inflammation by expressing a wide variety of receptors including pattern recognition receptors (PRRs) and cytokine receptors,^13^, ^14^ through which astrocytes can respond to multiple cues in the CNS microenvironment and produce various pro‐inflammatory cytokines and chemokines.^15^, ^16^ As a consequence, the astrocytes‐activated inflammation is closely linked with pathogenesis of neurodegenerative diseases or severe functional loss following CNS injury.^17^, ^18^, ^19^, ^20^, ^21^ Accumulative evidence has emphasized that the reactive astrocytes per se are major source of DAMPs following SCI, which interact with diverse cell types and contribute to crosstalk between immune and neural systems.^22^, ^23^ So far, less information is available regarding the regulatory mechanism of DAMPs production from astrocytes following SCI.

Macrophage migration inhibitory factor (MIF), a critical proinflammatory cytokines, is constitutively or inducibly expressed by a variety of cell types in most tissues, such as macrophages, fibroblasts, insulin secreting β‐cells of the pancreas, pituitary cells, and endothelial cells.^24^, ^25^ It has been shown to function in organ development, tumorigenesis, tissue injury, and inflammatory activation.^25^ In the spinal cord, expression of MIF is upregulated within neurons, microglia, and astrocytes after injury.^22^, ^26^ The overproduced cytokine activates microglia and astrocytic inflammation, resulting in a worsened lesion milieu.^26^, ^27^ Deletion or inhibition of MIF significantly attenuates local neuronal death and promotes functional recovery after SCI.^28^, ^29^ Thus, the elevated extent of MIF at the lesion site is proportional with the prognosis of CNS damage severity.^30^ Mechanistically, MIF performs proinflammatory function through binding to CD74 and/or CXCR2/4 receptors, and activates intracellular MAPKs and NF–κB signal pathway.^31^ However, the regulators responsible for the astrocytic production of MIF following SCI remain elusive.

Hypoxia induced by SCI always triggers many cellular responses.^32^, ^33^ The hypoxia‐inducible factors (HIFs) are oxygen‐dependent transcriptional activators in mediating various physiological and pathological processes, including inflammation and cancer by controlling a great of target genes.^34^, ^35^, ^36^ As heterodimers, HIFs are composed of an oxygen‐regulated α subunit (HIF‐1α, HIF‐2α, and HIF‐3α), and a constitutively expressed β subunit (HIF‐β).^37^, ^38^, ^39^ Lines of evidence demonstrate that functions of HIF‐1α and HIF‐2α vary depending on the cell type of tissue or tumor.^40^ Following SCI, a local hypoxic environment can induce overexpression of HIF‐1α, which in turn triggers a cascade of downstream signals. Accumulation of HIF‐1α at the lesion site of CNS has been shown to promote neuronal cell death and activate inflammation,^41^, ^42^ and suppression of HIF‐1α remarkably decreases the transition of reactive astrocytes and inflammatory activation in a rodent ischemia model.^43^, ^44^, ^45^, ^46^ Given that HIF‐1α acts as a universal switch in the regulation of inflammation, it is conceivable that HIF‐1α possibly promotes astrocytic production of MIF following rat SCI. In the present study, we analyzed the relations between HIF‐1α activation and MIF protein level in the astrocytes after rat spinal cord contusion. We further investigated the mechanism of hypoxia‐mediated MIF expression in astrocytes, and observed the effects of HIF‐1α suppression on the hindlimb motor functional recovery. Our results provided a prospective strategy for clinical treatment of SCI‐induced neuroinflammation.

## 2 MATERIALS AND METHODS

### 2.1 Animals

Adult male Sprague–Dawley (SD) rats weighing 180–220 g were provided by the Center of Experimental Animals, Nantong University. All the rats were housed in standard cages (five rats in each cage) under identical conditions maintained at 22 ± 2°C on a 12–12 h light–dark cycle and allowed ad libitum to feed and water. All animal protocols were approved by the *Animal Care and Use Committee of Nantong University* and the *Animal Care Ethics Committee of Jiangsu Province*.

### 2.2 Establishment of the SCI model and drug treatment

The number of animals subjected to surgical treatment was six per group in triplicate. The contusion SCI rat model was prepared as previously described.^47^ In short, all animals were anesthetized by intraperitoneal injection of sodium pentobarbital (30 mg/kg). The fur of the surgical area on the back was shaved and the skin was disinfected with chlorhexidine solution. A midline incision was made over the thoracic vertebrae, followed by a T9 laminectomy with the dura remaining intact. Using the IH‐0400 Impactor device (Precision Systems and Instrumentation), animals received a 150‐kilodyne contusion injury on the exposed spinal cord segment. The impact rod was removed immediately, and the wound was irrigated. The muscle layers and the skin were then sutured using silk threads. The procedure was visually checked by the formation of hematoma and by paralysis of the hindlimbs after animal awakening from the anesthesia. For drug delivery, a total of 8 μL of 8 mM HIF‐1α inhibitor LW6 (MCE) was slowly injected intrathecally, prior to the incision suture. The rats were undergone the proper care following surgery.

### 2.3 Cell culture and treatment

Astrocytes were prepared from the spinal cord of neonatal (1 day after birth) SD rats. The method of isolating and culturing astrocytes was according to the previous description.^29^ Briefly, the spinal cord segments were enzymatically dissociated in 0.25% trypsin (Gibco–BRL) for 15 min at 37°C, and the suspension was then centrifuged at 1200 rpm for 5 min. Finally, the cells were cultured in Dulbecco's modified Eagle's medium (DMEM) supplemented with 10% fetal bovine serum (FBS) and 1% penicillin/streptomycin in a humidified 5% CO_2_ incubator at 37°C. A monolayer of astrocytes was obtained 7–10 days after the plating. Microglia and other non‐astrocytes were separated from the bottom of the dishes by shaking and removed by changing the medium. Prior to the experiment, the second or third passage cells were kept quiescence in the medium containing 5% FBS for 4 days. Astrocyte phenotype was evaluated by positive staining of astrocytic marker glial fibrillary acid protein (GFAP). For treatment under hypoxic conditions, dish or plates were placed into a modified incubator chamber (Billups Rothenberg, Del Mar, CA), filled with 1% O_2_, 5% CO_2_, and 94% N_2_ (called hypoxic conditions) to culture. For CoCl_2_ or DMOG treatments, astrocytes were incubated with 0.5 mM CoCl_2_ or 1.5 mM DMOG at 37°C for desired time under normoxic condition.

### 2.4 Western blotting

Protein was extracted from cells or 0.5 cm spinal segments at the lesion site with a buffer containing 1% SDS, 100 mM Tris–HCl, 1 mM PMSF, and 0.1 mM β‐mercaptoethanol. Protein concentration of each specimen was detected by the BCA kit (Beyotime) to maintain the equivalent loads. Protein extracts were boiled for 5 min, and electrophoretically separated on 10% SDS–PAGE before electro‐transferred to PVDF membranes. The membranes were then subjected to the reaction with primary antibodies (1:1000 dilution) in TBS buffer at 4°C overnight, followed by a reaction with the secondary antibody conjugated with goat anti‐rabbit or goat anti‐mouse HRP (1:1000, Proteintech Group, Inc.) at room temperature for 2 h. The signaling of HRP activity was detected by an ECL kit (Vazyme). The membranes were scanned with a Tanon‐5200 Chemiluminescent Imaging System (Tanon), and the data were analyzed using PDQuest 7.2.0 software (Bio‐Rad). Antibodies used in Western blot were: β‐actin (1:5000; Proteintech), HIF‐1α (1:1000; Cell Signaling Technology), HIF‐2α (1:1000; Abcam), MIF (1:1000; Abcam), GAPDH (1:5000; Proteintech), and Histone H3 (1:1000; Proteintech).

### 2.5 Immunohistochemistry

The spinal segments or the cultured cells were harvested and fixed. The samples were incubated with S100β antibody (1:500 dilution; Sigma‐Aldrich), HIF‐1α (1:100 dilution; Abcam), monoclonal MIF antibody (1:100 dilution; Abcam), NeuN (1:200 dilution; Abcam), rabbit anti‐IBA‐1 (1:400 dilution; Wako), MBP (1:500; Cell Signaling Technology), or GFAP antibody (1:200 dilution; Abcam) at 4°C for 24 h. Then, the samples were further incubated with Cy3‐labeled goat anti‐rabbit or anti‐mouse secondary antibody (1:400 dilution; Abcam), or FITC‐labeled donkey anti‐rabbit or anti‐mouse secondary antibody (1:400 dilution; Abcam) at 4°C for 18 h. The images were obtained by using Leica TCS SP5 confocal laser scanning microscope system (Leica).

### 2.6 Quantitative real‐time polymerase chain reaction (qRT‐ PCR)

Total RNA was prepared with Trizol (Thermo Fisher Scientific) from cultured cells as mentioned above. The first‐strand cDNA was synthesized using HisScript II Q Select RT SuperMix for qPCR (R223‐01; Vazyme) in a 20 μL reaction system that contained 2 μg total RNA. The PCR reactions were performed in a final volume of 20 μL according to protocol of ChamQTM SYBR qPCR Master Mix kit (Q711‐02; Vazyme, Nanjing, China). The primers used in the reactions were designed based on the genome sequences with sense primer 5′‐GGT CAC ACC GCA CTT AAC AC‐3′ and anti‐sense primer 5′‐CGC TCG TGC CAC TAA AAG TC‐3′ for MIF. The LightCycler96 software (Roche) was used for PCR reactions and the data analysis.

### 2.7 ELISA

Tissue samples were sonicated using the lysis buffer with protease inhibitor PMSF as mentioned above. The lysis was centrifuged at 12,000 rpm for 15 min at 4°C, and the supernatant was collected for MIF (Elabscience) ELISA assay. The concentration of MIF was expressed as ng/mg for the lysate of the cord tissues. Plates were read with a multifunctional enzyme marker (Biotek Synergy2) at a 450 nm wavelength.

### 2.8 Subcellular fractionation

After the cell stimulation with DMOG (1.5 mM) for 4 h, the extraction of cytosolic and nuclear protein from astrocytes was isolated according to the protocol of Nuclear and Cytoplasmic Protein Extraction Kit (Beyotime). Briefly, the cells were washed with ice‐cold PBS, followed by lysis in 200 μL cytoplasmic protein extraction agent A with 1 mM PMSF on ice for 15 min after a high‐speed vortex for 5 s. Then, the cytoplasmic protein extraction agent B was added, and the mixture was vortexed for 5 s prior to incubation on ice for 1 min. Samples were centrifuged at 16,000 *g* at 4°C for 5 min, and the supernatant (cytosolic fraction) was immediately collected. The pellet (nuclear fraction) was resuspended in nuclear protein extraction agent supplemented with 1 mM PMSF. The samples were vortexed thoroughly at high speed for 15–20 times at 30 min, followed by a centrifugation at 16,000 *g* for 10 min. Then, the supernatants containing the nuclear extracts were collected. Proteins of nuclear and cytosolic extracts were determined by Western blot. Both GAPDH and Histone H3 were used as internal control of cytoplasmic and nuclear proteins, respectively.

### 2.9 Luciferase assays

The sequence of MIF promoter was amplified by PCR using primers: 5′‐ TTT CTC TAT CGA TAG GTA CCT TCA GGT ACC TGG GGA GTC C‐ 3′; 5′‐ CTT AGA TCG CAG ATC TCG AGA GCT CAG GAC CGC CCC AAG CCG G‐ 3′. The sequence at length of 2040 bp was cloned into pGV238 reporter vector to form a reporter plasmid (MIF‐wt), and a control plasmid (MIF‐mut) was constructed by mutation of A‐T at predicted HIF‐1α binding elements. Cells grown to 70% confluence were transfected in triplicate with pGV238‐MIF‐wt, pcDNA‐HIF‐1α or pGV238‐MIF‐mut. After 48 h of transfection, cells were collected and the luciferase activity was measured using the Luciferase Reporter Assay System (Promega) according to the manufacturer's protocol.

### 2.10 Behavioral tests

The hindlimb locomotor function recovery was evaluated using the Basso, Beattie, and Bresnahan (BBB) locomotor scale. After intrathecal injection of 8 μL of DMSO or 8 μL of 8 mM LW6 at 0, 7, 14, and 21 days, three well‐trained investigators blind to the study were invited to observe the behavior of rats for 5 min. The BBB score ranged from 0 to 21 according to the rating scale. Every rat had a BBB score of 21 before surgery, and 0 to 1 after a successful SCI.

### 2.11 Statistical analysis

Statistical analysis used GraphPad Prism 8 software. All data are presented as mean ± SEM. The D'Agostino‐Pearson omnibus normality test was used to assess data distribution. Comparisons between two groups with normal distribution were analyzed by two‐tailed unpaired Student's *t* test or the Mann–Whitney test when the distribution was not parametric. Differences between multiple groups were analyzed using one‐way or two‐way analysis of variance, followed by Tukey's or Sidak's post hoc test. *P*‐value <0.05 was considered statistically significant and was denoted in the figures as *P* < 0.05.

## 3 RESULTS

### 3.1 SCI significantly induces astrocytic expression of HIF‐1α

**FIGURE 1:**
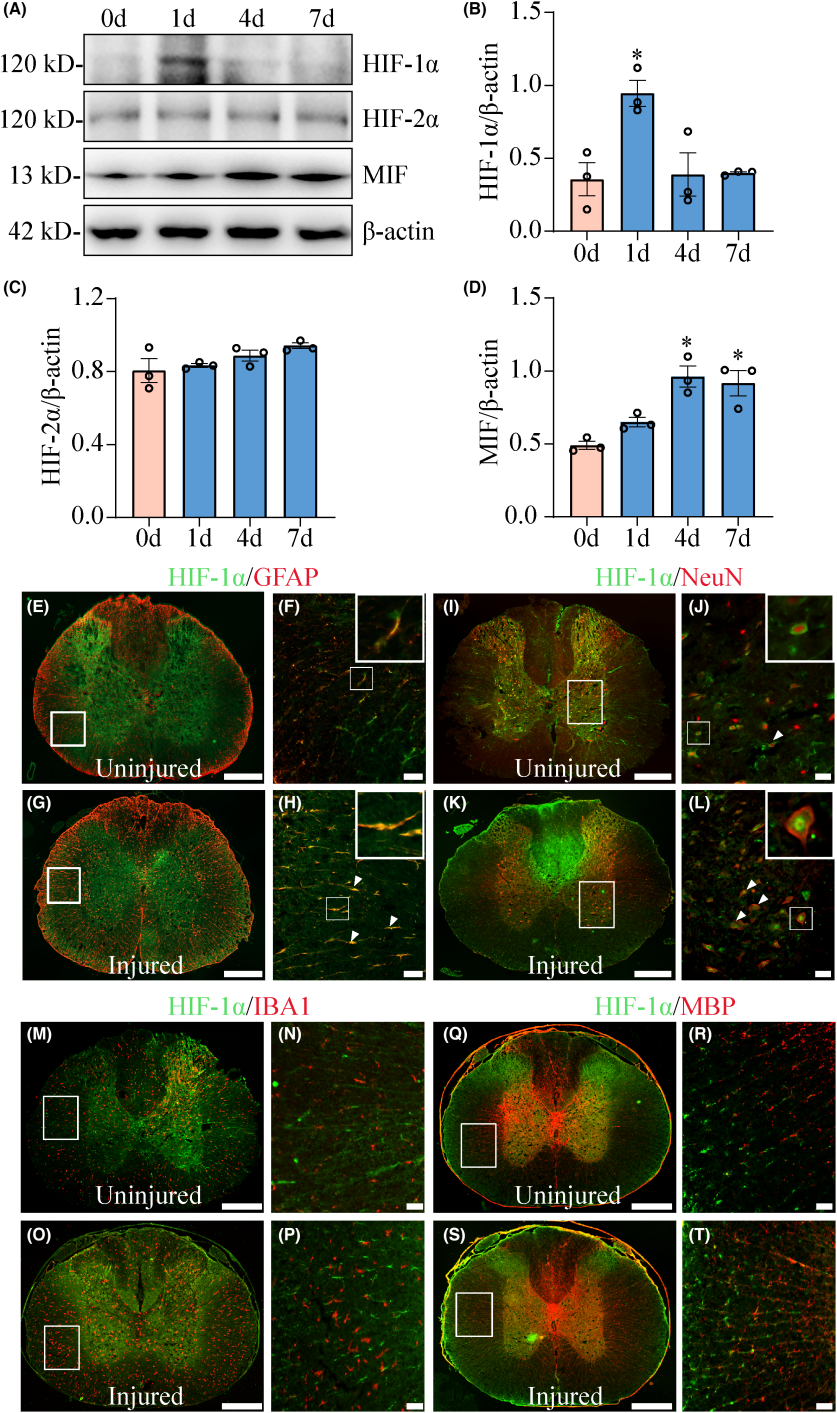
Determination of HIF‐1α and MIF expression at the lesion sites following SCI. (A) Western blot analysis of HIF‐1α, HIF‐2α and MIF protein following SCI at 0, 1, 4, and 7 days, respectively. Quantities were normalized to an endogenous β‐Actin. (B–D) Quantification data as shown in (A). (E–T) Colocalization of HIF‐1α with GFAP‐positive astrocytes, NeuN‐positive neurons, IBA‐positive microglia, and MBP‐positive oligodendrocytes at 0d (uninjured) and 1d (injured). The rectangle indicates the magnified region. Arrowheads indicate the colocalization signals. Experiments were performed at least in triplicate. Data are expressed as mean ± SEM, *p < 0.05. Scale bars, 500 μm, and 50 μm in magnification.

To understand the potential effect of hypoxia on SCI‐induced dynamic changes of MIF expression, the protein levels of HIF‐1α and HIF‐2α at lesion sites were determined at 0, 1, 4, and 7 days following spinal cord contusion. Western blot demonstrated that the expression of HIF‐1α, but not of HIF‐2α, was significantly induced at 1 day after SCI (Figure 1A–D). Meanwhile, the protein levels of MIF were also elevated at 4 and 7 days (Figure 1B,D), indicating SCI‐induced hypoxia and overproduced DAMPs around the lesion milieu.

To examine specific cell types that were induced to express HIF‐1α in the injured cord, tissue sections were prepared from 0.5 cm segments harvested around the epicenter of contusion at 1d following SCI. Immunostaining revealed that HIF‐1α protein colocalized with GFAP‐positive astrocytes (Figure 1E–H) and NeuN‐positive neurons (Figure 1I–L), rather than with IBA1‐positive microglia (Figure 1M–P) or MBP‐positive oligodendrocytes (Figure 1Q–T) before or after SCI. The data indicate that the astrocytes, together with neurons, are the hypoxia‐sensitive cell types following SCI.

**FIGURE 2:**
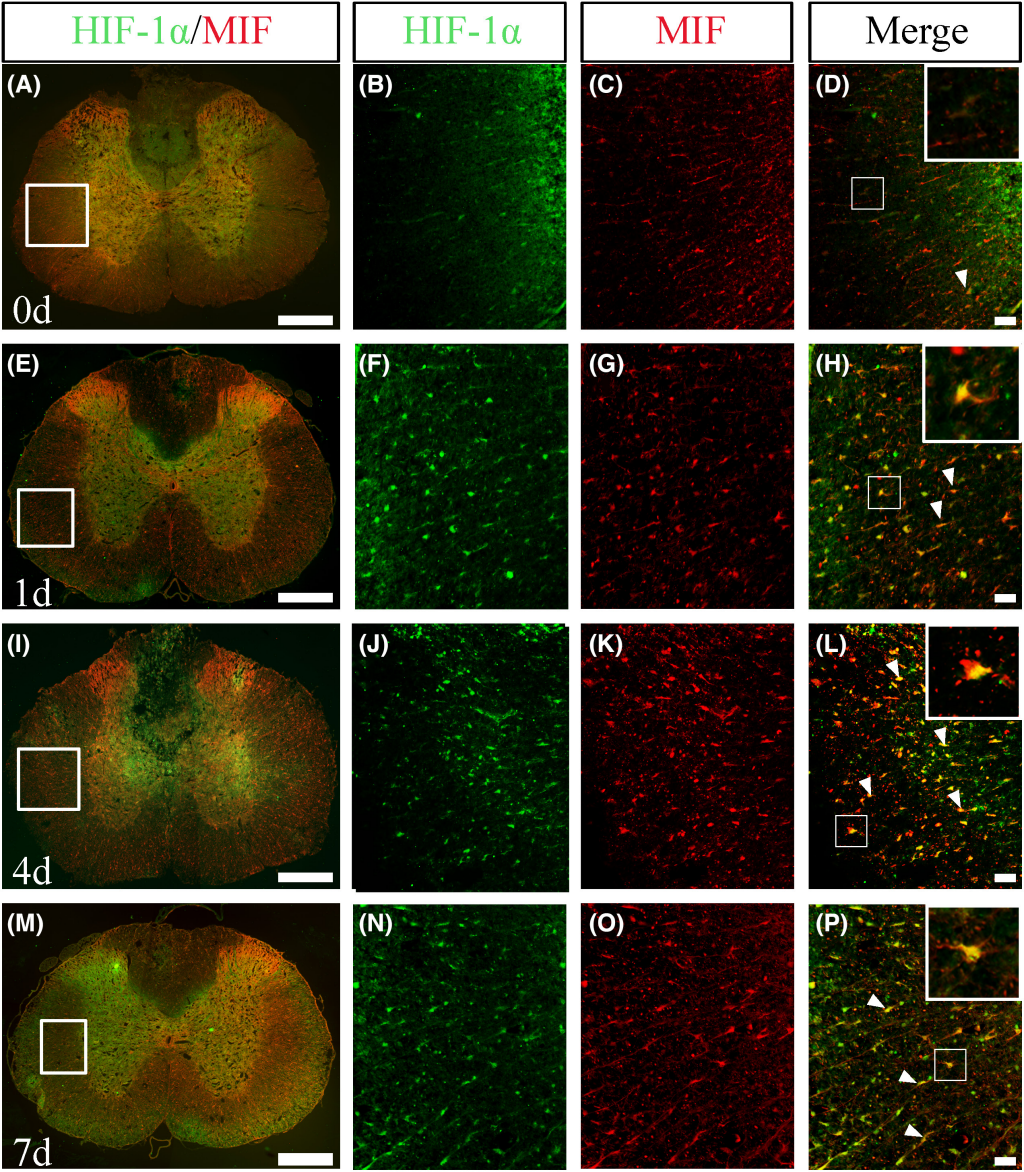
Analysis of HIF‐1α colocalization with MIF in the cord tissues. Sections were prepared at 0, 1, 4, and 7 days following SCI. The rectangle indicates the magnified region. Arrowheads indicate the colocalization signals. Scale bars, 500 μm in (A, E, I, M); 50 μm in magnifications.

To observe whether SCI‐induced HIF‐1α expression had a colocalization with those of MIF, immunostaining was performed on the cord at lesion sites following SCI. Results revealed that HIF‐1α and MIF positive signals were well colocalized in the cord tissues, and significantly induced by SCI at 4 and 7 days after SCI (Figure 2A–P). The data indicate that the SCI‐induced HIF‐1α at the lesion sites may be involved in regulation of MIF expression.

### 3.2 Hypoxia promotes astrocytic expression of MIF

**FIGURE 3:**
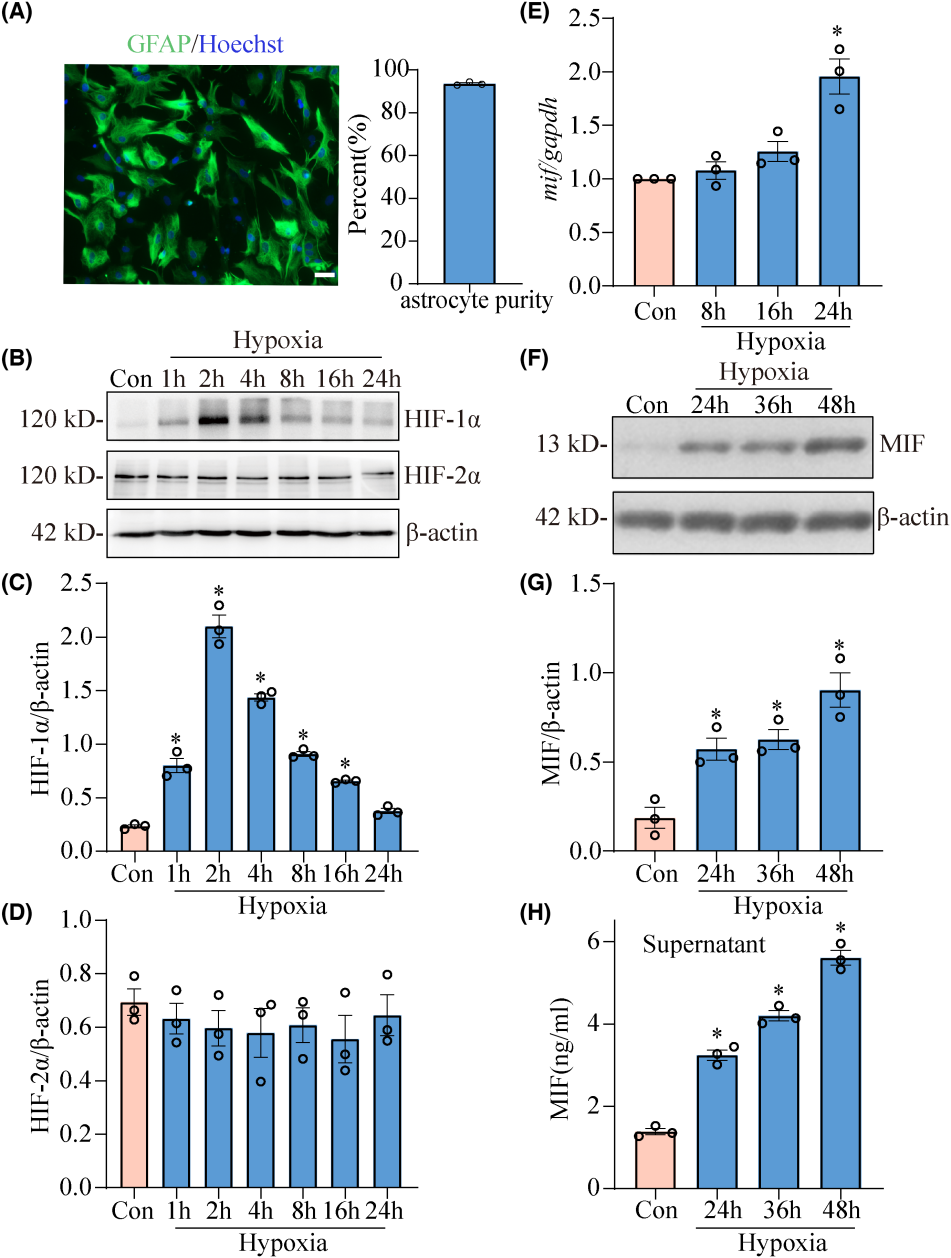
Effects of hypoxia on the astrocytic expression of MIF. (A) Primary cultured astrocytes were stained with GFAP and Hoechst 33342, showing purity over 92%. (B) Western blot analysis of HIF‐1α and HIF‐2α protein levels in the astrocytes induced by hypoxia for 0–24 h. (C, D) Quantification data as shown in (B). Quantities were normalized to endogenous β‐Actin. (E) RT–PCR analysis of *mif* in the astrocytes induced by hypoxia for 8, 16, and 24 h. Quantities were normalized to endogenous *gapdh*. (F) Western blot analysis of MIF protein levels in the astrocytes induced by hypoxia for 24, 36, and 48 h, respectively. (G) Quantification data as shown in (F). (H) ELISA determination of MIF in the supernatants of cultured astrocytes exposed to hypoxia for 24, 36, and 48 h. Experiments were performed in triplicates. Error bars represent the SEM (*p < 0.05).

To validate the inducible roles of hypoxia on the production of MIF in astrocytes, the primary astrocytes isolated from spinal cord were cultured with purity over 92% (Figure 3A). The cells were incubated in an anoxic incubator with 1% oxygen for different times, and then collected for analysis. Western blot assay showed that the protein level of HIF‐1α was significantly increased from 1 h with a peak at 2 h following hypoxia treatment, but that of HIF‐2α was unaffected (Figure 3B–D). The expression of MIF at transcriptional and translational level was determined following cell hypoxia for 8 to 48 h. The hypoxia stress promoted MIF transcription within astrocytes at 24 h, as examined by PCR assay. ELISA measurement of both cell lysates and supernatants demonstrated that astrocytes were induced to produce MIF in response to hypoxia for 24, 36, and 48 h (Figure 3E–H). These data indicate that hypoxia promotes MIF production from astrocytes.

### 3.3 Activation of HIF‐1α facilitates the astrocytic production of MIF

**FIGURE 4:**
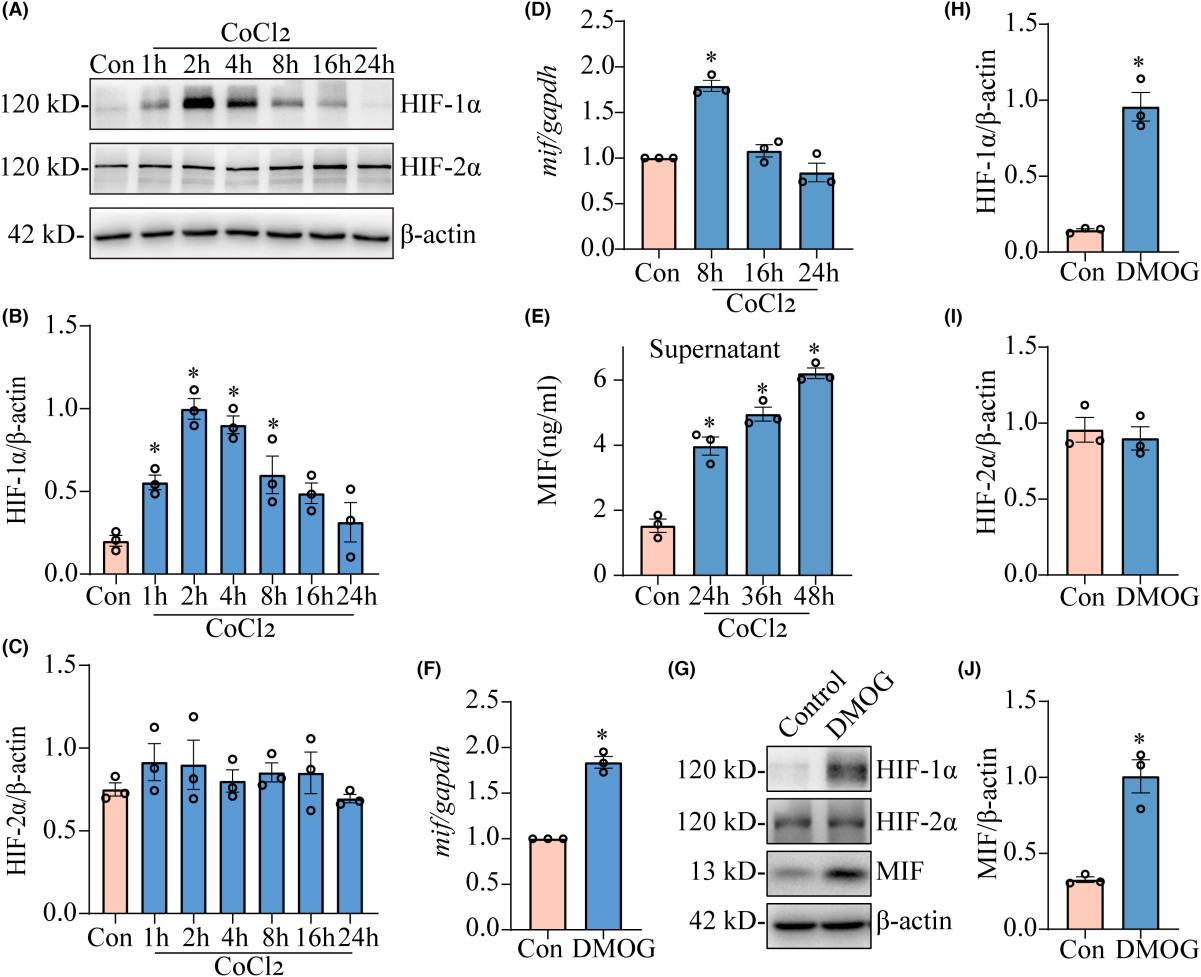
Effects of HIF‐1α agonist CoCl_2_ and DMOG on the production of astrocytic MIF. (A) Western blot analysis of HIF‐1α and HIF‐2α protein levels in the astrocytes stimulated by 0.5 mM CoCl_2_ for 0–24 h. (B, C) Quantification data as shown in (A). Quantities were normalized to endogenous β‐Actin. (D) RT‐PCR analysis of *mif* in the astrocytes stimulated by 0.5 mM CoCl_2_ for 8, 16, and 24 h. Quantities were normalized to endogenous *gapdh*. (E) ELISA determination of MIF in the supernatants of cultured astrocytes stimulated by 0.5 mM CoCl_2_ for 24, 36, and 48 h. (F) RT–PCR analysis of *mif* in the astrocytes stimulated by 1.5 mM DMOG for 24 h. Quantities were normalized to endogenous *gapdh*. (G) Western blot analysis of HIF‐1α, HIF‐2α, and MIF protein levels in the astrocytes stimulated by 1.5 mM DMOG for 24 h. (H–J) Quantification data as shown in (G). Experiments were performed in triplicates. Error bars represent the SEM (*p < 0.05).

To elucidate whether hypoxia‐activated HIF‐1α participates in the astrocytic production of MIF, cobalt chloride (CoCl_2_), the chemical inducer of HIF‐1α, was applied to stimulate astrocytes. As was expected, cell exposure to 0.5 mM CoCl_2_ significantly promoted the transcription and translation of MIF in the astrocytes, by activation of HIF‐1α, but not of HIF‐2α (Figure 4A–E). Similarly, cell treatment with 1.5 mM dimethyloxalylglycine (DMOG), another HIF‐1α activator, also resulted in the induction of MIF in the astrocytes, together with activation of HIF‐1α (Figure 4F–J). The data indicate that activation of HIF‐1α facilitates MIF production from astrocytes.

### 3.4 HIF‐1α selective inhibitor serves to attenuate the astrocytic production of MIF

**FIGURE 5:**
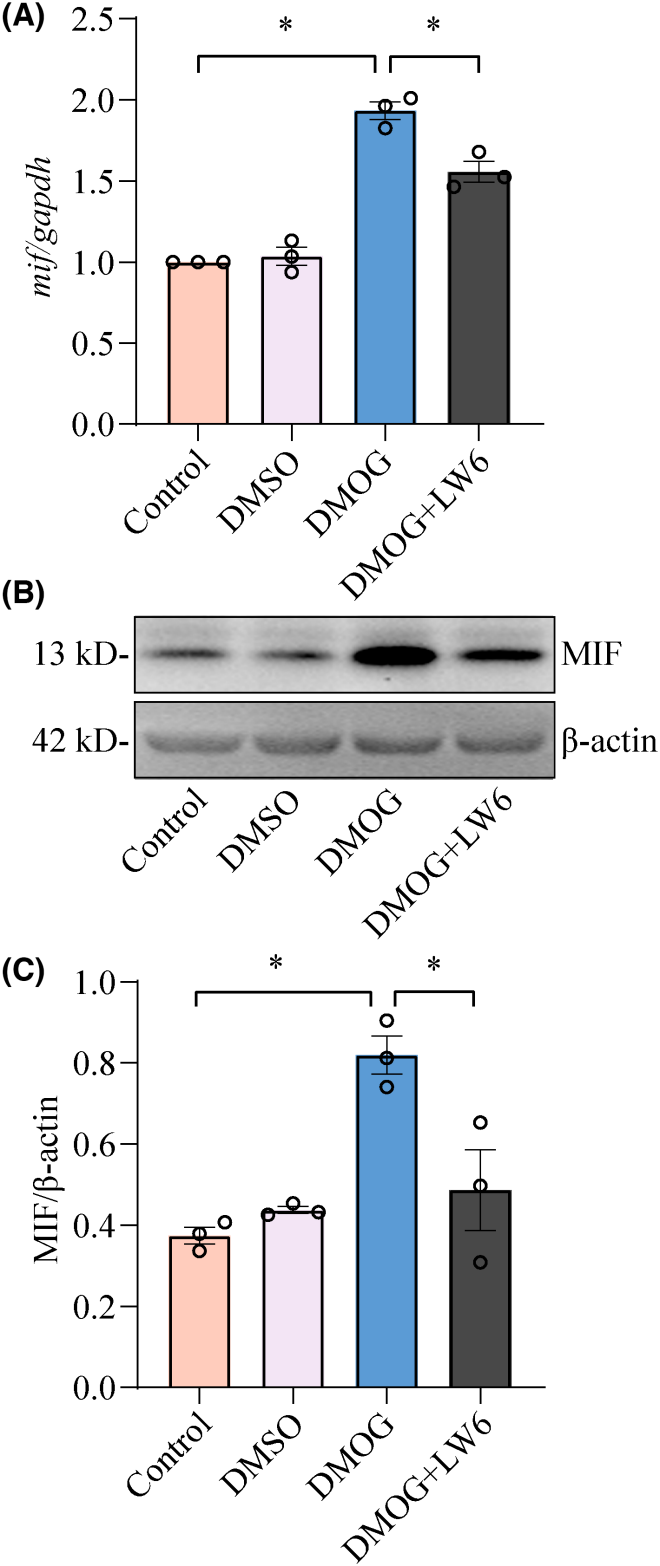
HIF‐1α inhibitor attenuates MIF production of astrocytes. (A) RT‐PCR analysis of *mif* in the astrocytes stimulated by 1.5 mM DMOG in the presence or absence of 20 μM LW6 for 24 h. Quantities were normalized to endogenous *gapdh*. (B) Western blot analysis of MIF protein levels in the astrocytes treated by 20 μM LW6 in the presence of 1.5 mM DMOG. (C) Quantification data as shown in (B). Quantities were normalized to the endogenous β‐Actin. Experiments were performed in triplicates. Error bars represent the SEM (*p < 0.05).

To clarify whether inhibition of HIF‐1α can attenuate the astrocytic production of MIF, a selective inhibitor LW6 at final concentration of 20 μM was added to culture medium of astrocytes in the presence of 1.5 mM DMOG. PCR results showed that the transcription of MIF in the astrocytes was markedly inhibited by LW6 at 24 h (Figure 5A). Also, the protein levels of MIF were decreased after treatment of astrocytes with LW6 in the presence of DMOG (Figure 5B,C). The data indicate that inhibition of HIF‐1α is able to suppress hypoxia‐mediated astrocytic production of MIF.

### 3.5 HIF‐1α regulates MIF production by interaction with the promoter of MIF

**FIGURE 6:**
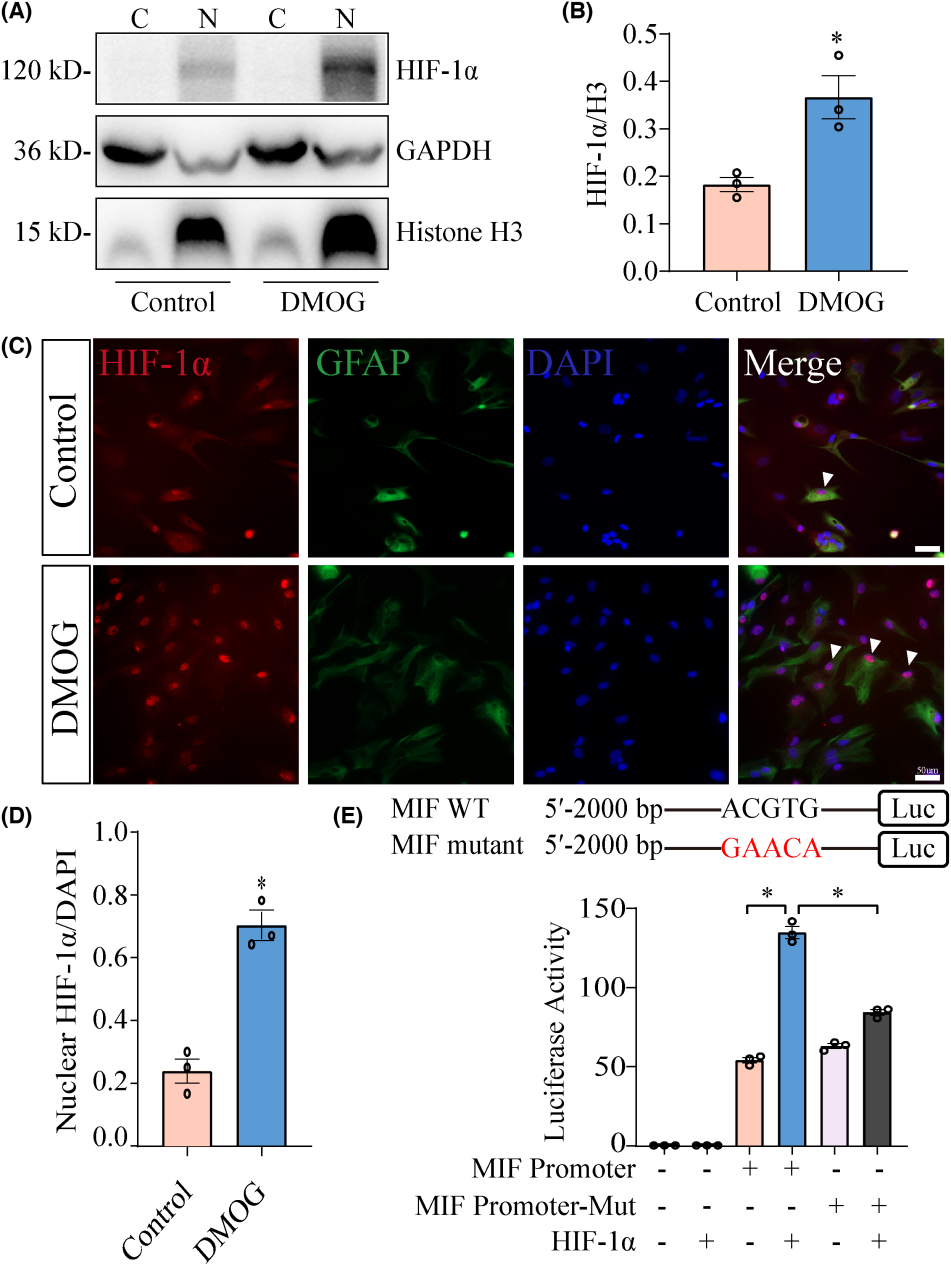
HIF‐1α promotes astrocytic expression of MIF through interaction with the promoter. (A) Western blot analysis of HIF‐1α protein levels in the cytoplasm and nucleus following astrocytes treatment with 1.5 mM DMOG for 4 h. Quantities were normalized to endogenous GAPDH (cytoplasm) and Histone H3 (nucleus). (B) Quantification data as shown in (A). (C) Colocalization of HIF‐1α with GFAP and DAPI in the astrocytes treated by 1.5 mM DMOG for 4 h. Arrowheads indicate the colocalization signals. (D) Quantification data as shown in (C). (E) Assay of HIF‐1α overexpression on the activity of luciferase in MIF‐promoter constructs. Scale bar, 50 μm in (C). Experiments were performed in triplicates. Error bars represent the standard deviation (*p < 0.05).

Previous studies have shown that HIF‐1α regulates expression of downstream genes through entering into the cellular nuclei and binding to the promoter of the target genes. To shed light on the regulatory mechanism of HIF‐1α on the astrocytic MIF, the subcellular distribution of HIF‐1α was firstly examined following astrocyte treatment with 1.5 mM DMOG for 4 h. Immunoblot analysis for the cytosolic and nuclear fraction showed that HIF‐1α was predominantly distributed in the nucleus of astrocytes rather than in the cytoplasm in response to DMOG stimulation (Figure 6A,B). Immunostaining further confirmed the nuclear translocation of HIF‐1α following DMOG‐induced hypoxia (Figure 6C,D). In addition, luciferase assays were performed to detect the activity of luciferase reporter gene in the MIF‐promoter constructs. The results revealed that overexpression of HIF‐1α significantly increased the activity of luciferase in MIF‐promoter constructs, whereas in MIF‐promoter‐Mut did not (Figure 6E). The data indicate that HIF‐1α regulates MIF expression by interaction with MIF promoter in the astrocytes.

### 3.6 Inhibition of HIF‐1α significantly attenuates the astrocytic production of MIF following SCI

**FIGURE 7:**
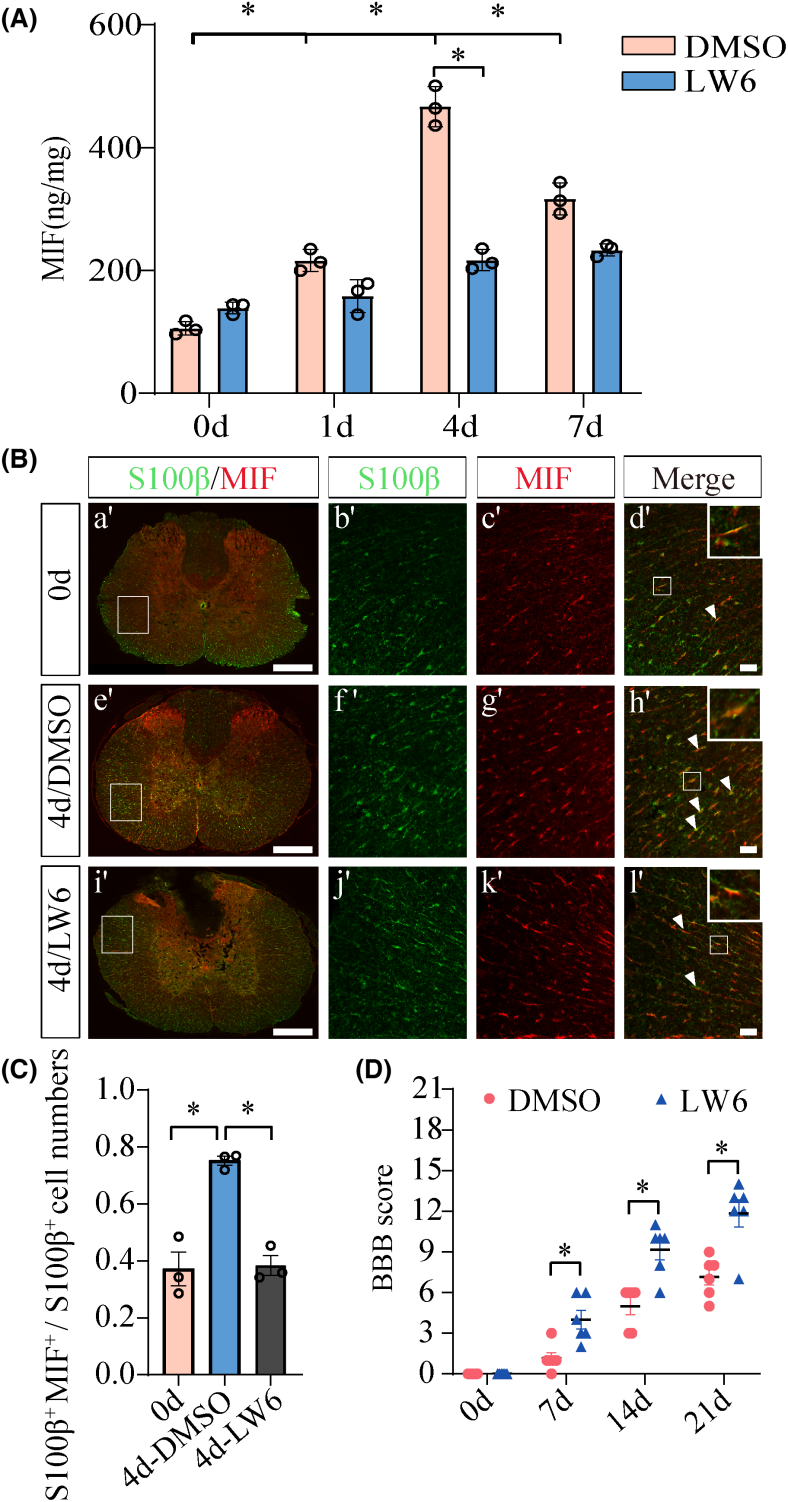
HIF‐1α inhibitor attenuates MIF production at lesion site of the cord and evaluation of rat hindlimb motor function following SCI. (A) ELISA assay of MIF at lesion sites following intrathecal injection with 8 μL of 8 mM LW6 for 0, 1, 4, and 7 days, respectively. (B) Immunostaining showed colocalization of MIF with S100β‐positive astrocytes following injection of 8 μL of 8 mM LW6 for 0 and 4 days. Rectangles indicate the magnified region. Arrowheads indicate the positive signals. (C) Quantification data as shown in (B). (D) BBB score of hindlimb at 0, 7, 14 and 21 days following injection of 8 μL of 8 mM LW6 at lesion sites, *n* = 6. Scale bars, 500 μm in (a′), (e′), and (i′); 50 μm in magnifications. Experiments were performed in triplicates. Error bars represent the standard deviation (*p < 0.05).

To examine the effects of HIF‐1α inhibition on the production of MIF following SCI, a total of 8 μL of 8 mM LW6 were intrathecally injected at lesion sites. ELISA assay for the 0.5 cm cord segments demonstrated that the protein levels of MIF were significantly reduced at lesion site following LW6 administration at 4 days (Figure 7A). Immunofluorescence showed that the expression of MIF in the astrocytes was markedly decreased by the LW6 treatment at 4 days (Figure 7B,C).

As massive release of MIF can result in the aggravation of neuroinflammation and severe loss of locomotor function, HIF‐1α inhibition‐mediated reduction of MIF following SCI was thus expected to have protective roles on the functional recovery. To evaluate the effects of LW6 on the contused spinal cord of rats, behavioral tests were performed during 3 weeks to assess hind‐limb motor function. BBB scores showed that administration of 8 μL of 8 mM LW6 at the lesion sites of the cord efficiently promoted the improvement of the locomotor function of rats in comparison with the control (Figure 7D). The data indicate that suppression of HIF‐1α is able to reduce the production of MIF in astrocytes, which in turn contributes to the locomotor functional recovery following SCI.

## 4 DISCUSSION

DAMPs, together with other proinflammatory mediators, are key activators of neuroinflammation.^27^, ^48^, ^49^, ^50^, ^51^ Various DAMPs, including several heat‐shock proteins, HMGB1, hyaluron fragments, ATP, uric acid, heparin sulfate, and the DNA materials, are recognized to aggravate neuropathology following CNS injury or disorder.^52^, ^53^ MIF is recently defined as one of DAMPs that mediates multiple neurological diseases and severity of SCI.^24^ Distinct to other DAMPs, the inducers of MIF production in the injured CNS have not been fully elucidated. We have previously shown that injury‐activated thrombin is able to contribute to the MIF production following SCI.^22^ In the present study, we revealed that HIF‐1α also served as an enhancer of MIF expression in astrocytes, suggesting that SCI‐induced production of MIF is promoted by multiple mediators. Whether other DAMPs are governed by HIF‐1α following SCI deserves further study.

Hypoxia is associated with neurological disorders, such as trauma, ischemic stroke, Alzheimer's disease (AD) and Parkinson's disease (PD).^33^, ^54^ Once the CNS, the most sensitive tissue to the supply of oxygen, is damaged, HIF‐1α is immediately induced by multiple cell types to regulate the transcription of various target genes that mediate oxidative stress, inflammation, cell death and adaptive response.^36^, ^55^, ^56^ For example, within the earliest 24–48 h after ischemia, the accumulation of HIF‐1α triggers a series of brain events related to pentose phosphate pathway inhibition and promotes neuronal apoptosis by upregulating the expression of downstream genes in neurons such as NKCC1 (Na^+^‐dependent chloride transporter 1) in rat.^42^, ^57^, ^58^ Notably, hypoxia can activate HIF‐1α within 1–3 h, whereas HIF‐1α‐mediated expression of target molecules, as well as related pathophysiological effects needs to take more time. Several controversy debates regarding to the roles of HIF‐1α following CNS insults, however, come from that a blockade of HIF‐1α activation can lead to increased brain damage and decreased neurogenesis during the postischemic stages both in vitro and in vivo.^59^, ^60^ In this context, HIF‐1α transduction signaling promotes neuronal survival and the proliferation of neuronal progenitor cells by increasing expression of erythropoietin (EPO) and CBX7 during the later stage.^61^, ^62^, ^63^, ^64^ Thus, HIF‐1α functions in neuropathology dependent on spatio‐temporal injury modes, which drives differential downstream targets. As for HIF‐1α‐mediated inflammation in the pathological CNS, HIF‐1α is found to promote the production of chemokines and proinflammatory cytokines including MCP‐1, MCP‐5, TNF‐α, and IL‐1β in hypoxic astrocytes, which aggravate the neuroinflammation after acute ischemic stroke.^65^, ^66^ In the present study, we showed that SCI‐induced HIF‐1α was able to facilitate proinflammatory MIF production from astrocytes, in consistent with those of previous documents, strengthening the importance of HIF‐1α in mediating neuropathology.

Four HIF subtypes (HIF‐1α, HIF‐1β, HIF‐2α, and HIF‐3α) have been shown to all express in the CNS. HIF‐1β is constitutively expressed as a nuclear protein, whereas HIF‐1α is ubiquitously expressed in most cell types with susceptibility to hypoxia stress.^67^ Stabilized HIF‐1α translocates into nucleus to form a heterodimer with HIF‐1β, and then binds to the hypoxia‐response element (HRE) within the promoter of hypoxia‐sensitive target genes.^68^, ^69^ Although HIF‐2α shares 48% identity with HIF‐1α and upregulates its expression in response to hypoxia within endothelial cells, but it fails to increase in hypoxia‐inducible rat brain.^70^, ^71^ Therefore, HIF‐1α, rather than HIF‐2α, acted as the prominent proinflammatory mediator in the insulted CNS. Such evidence was also validated by the present study, showing that HIF‐1α promoted astrocytic production of MIF following SCI. HIF‐3α is able to heterodimerize with HIF‐1β, but acts as a negative regulator of HIF‐1α in the context of hypoxia.^72^, ^73^, ^74^ Whether HIF‐3α is involved in the regulation of HIF‐1α following SCI remains clarified.

Inhibition of HIF‐1α by various inhibitors is effective in attenuating HIF‐1α‐mediated pathological events, such as activation of inflammation and invasion of tumors.^75^, ^76^, ^77^ LW6, an aryloxyacetylamino benzoic acid derivative, is able to efficiently inhibit the expression and accumulation of HIF‐1α.^78^, ^79^ The usage of LW6 in the colon cancer cells has been shown to promote the degradation of HIF‐1α by upregulation of VHL without affecting HIF‐1β expression, giving rise to competent anti‐tumor efficacy in vivo.^77^ In the present study, we displayed that LW6 significantly deceased the production of MIF by inhibition of HIF‐1α, which in turn promoted the functional recovery of rat hindlimb. As such, LW6 may hint a valuable pharmaceutical drug in clinical treatment of neuroinflammation.

## 5 CONCLUSIONS

**FIGURE 8:**
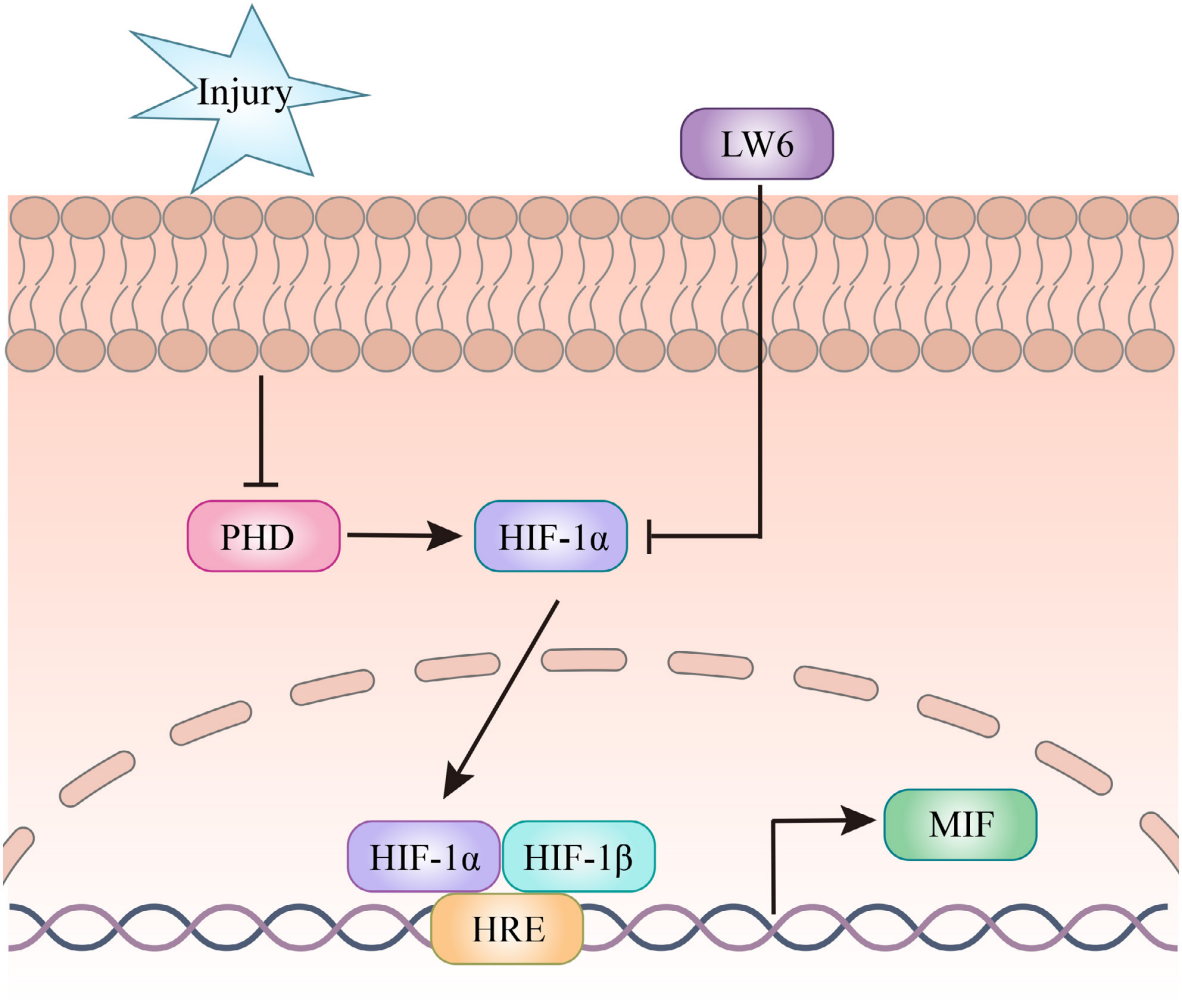
Illustration of HIF‐1α in promoting astrocytic production of MIF following SCI.

SCI‐induced activation of HIF‐1α is able to induce the astrocytic production of MIF through interaction with the promoter (Figure 8). Inhibition of HIF‐1α following SCI will reduce the protein levels of MIF at lesion site, which in turn favors for the recovery of rat locomotor function.

## AUTHOR CONTRIBUTIONS

YjunW and YjieW designed this work. YjieW wrote the article. HH performed the experiments. HH, YH, AL, LN, SL, BH, XZ, HS, RC, CY, YZ, YjunW, and YjieW analyzed the data. HH, YjunW, and YjieW joined discussions. All the authors have approved the present version of the manuscript and have agreed to be accountable for all aspects of the work regarding questions related to the accuracy or integrity of any part of the work.

## FUNDING INFORMATION

This research was funded by the National Natural Science Foundation of China (No. 31871211), the National Key Research and Development Program of China (No. 2020YFA0113600), the Priority Academic Program Development of Jiangsu Higher Education Institutions (PAPD), and the Nantong Science and Technology Project (No. JC2021180).

## CONFLICT OF INTEREST STATEMENT

The authors declare no conflict of interest.

## Supporting information


Appendix S1

## Data Availability

The datasets used and/or analyzed during the current study are available from the corresponding author on reasonable request.
