# Supplementary figures and images for: HIF‐1α promotes astrocytic production of macrophage migration inhibitory factor following spinal cord injury
Source: CNS Neurosci Ther. 2023 Jun 19;29(12):3802–14. doi: 10.1111/cns.14300 (PMC10651974; doi:10.1111/cns.14300)

Figure 1

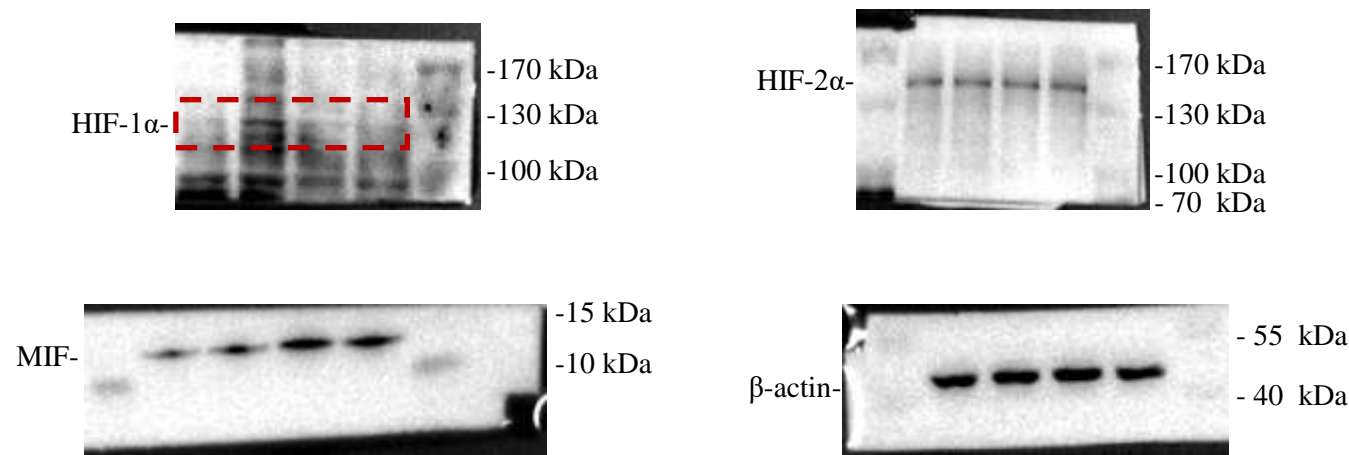

Figure 3b

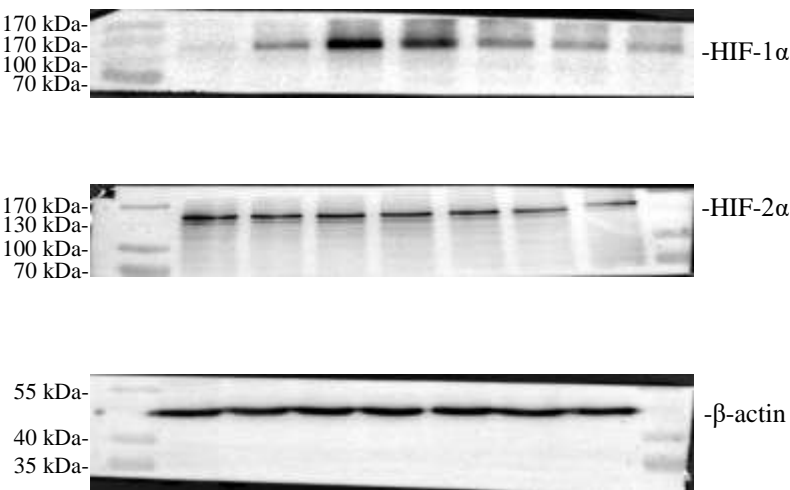

Figure 3f

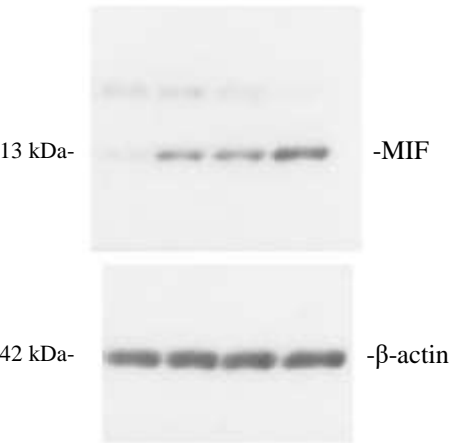

Figure 4a

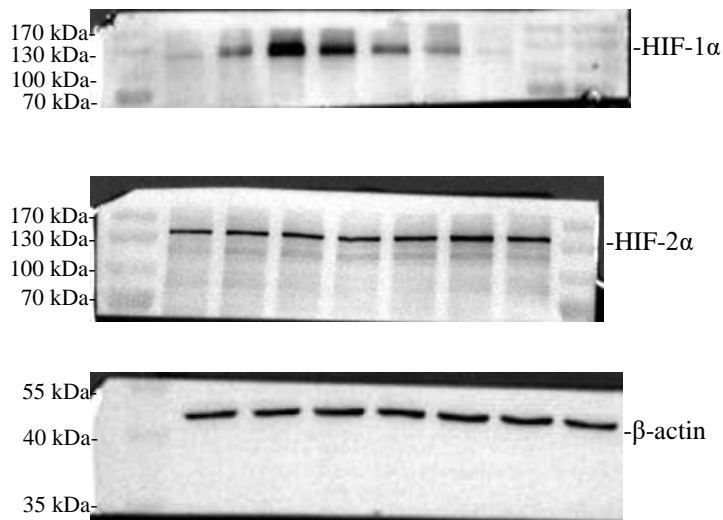

Figure 4g

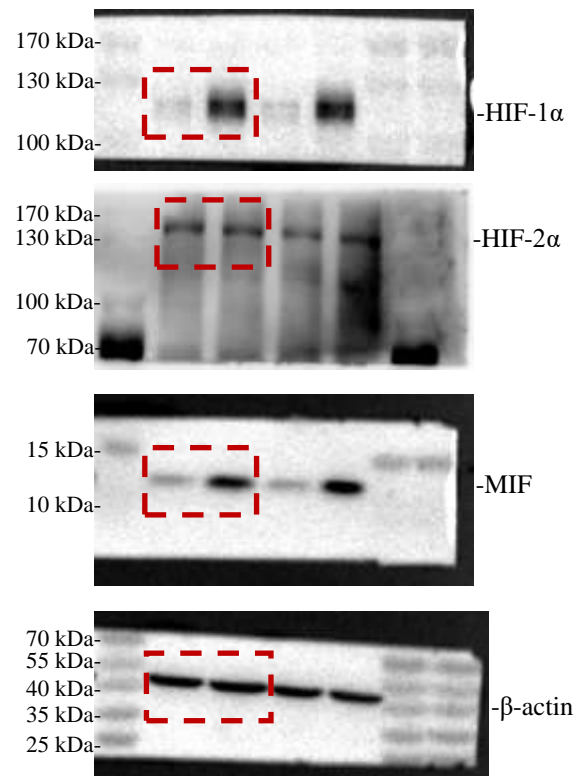

Figure 6

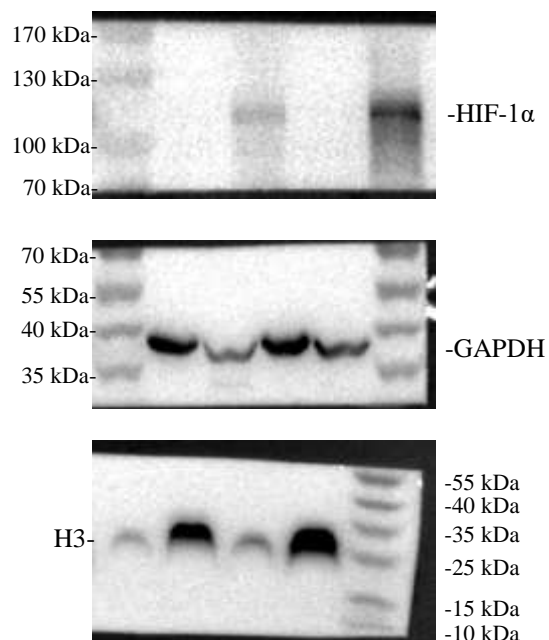

Figure 5

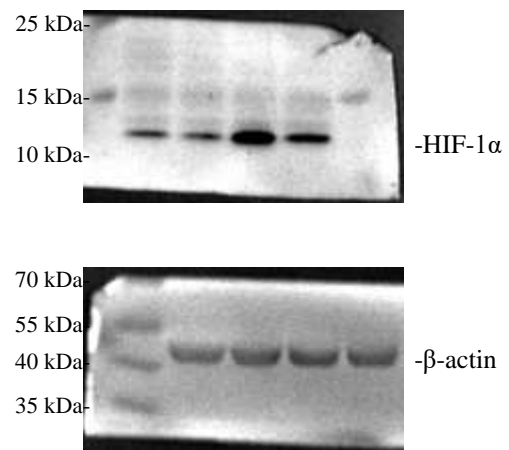

Supplement: Supplementary file 1 — Appendix S1 [file CNS-29-3802-s001.pdf]
